# Bile duct adenoma: a case report and literature review

**DOI:** 10.1186/1477-7819-12-125

**Published:** 2014-04-26

**Authors:** Lei Chen, Mai Yu Xu, Feng Chen

**Affiliations:** 1 Department of Hepatobiliary Surgery, Wenzhou Central Hospital, Da Jian Lane 32, Wenzhou, Zhejiang, China

## Abstract

**Background:**

Bile duct adenoma (BDA) is a comparatively rare disease clinically, therefore, there are relatively few reported cases about it both in China and abroad.

**Case presentation:**

Herein, we present a 51-year-old man, diagnosed preoperatively with enhanced-contrast abdominal computed tomography, as having a nodule in the left hepatic. The patient underwent a liver tumor resection, and the histological examination revealed bile duct adenoma (BDA).

**Conclusions:**

BDA is an extremely rare benign tumor, which is difficult to distinguish BDA from hepatocellular carcinoma definitely preoperatively, surgical resection is needed as a way of treatment.

## Background

Bile duct adenoma (BDA) is a comparatively rare disease clinically, therefore, there are relatively few reported cases about it both in China and abroad. Herein, we report a case of BDA as well as a literature review, thus presenting a preliminary analysis on BDA.

## Case presentation

**Figure 1:**
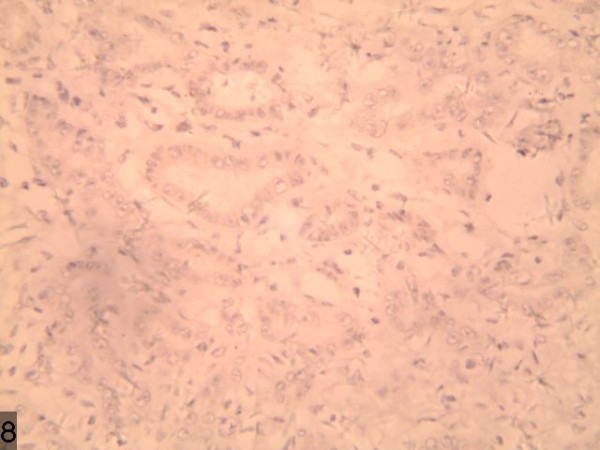
p53 stains were negative.

**Figure 2:**
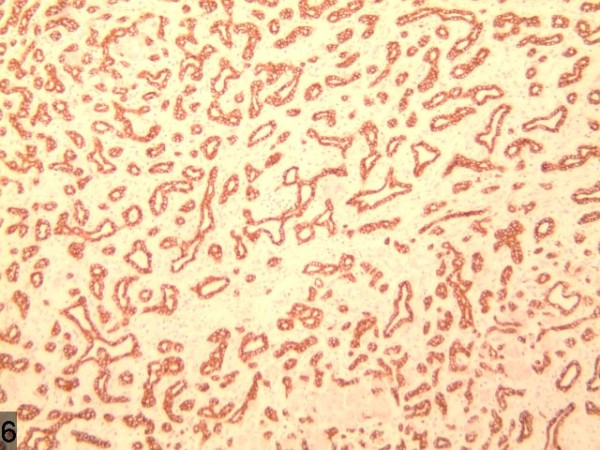
Strong reactivity for CK7.

**Figure 3:**
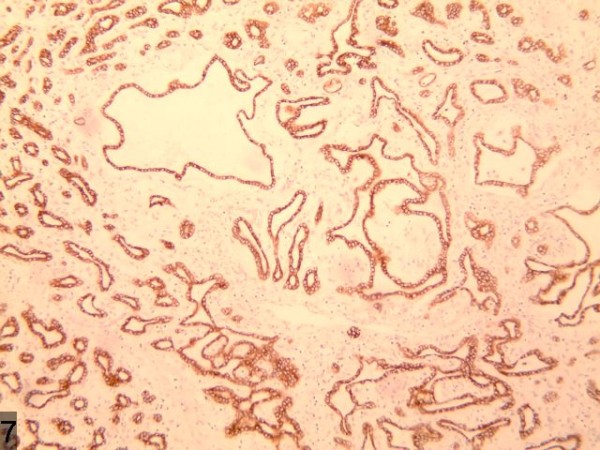
Strong reactivity for CK19.

**Figure 4:**
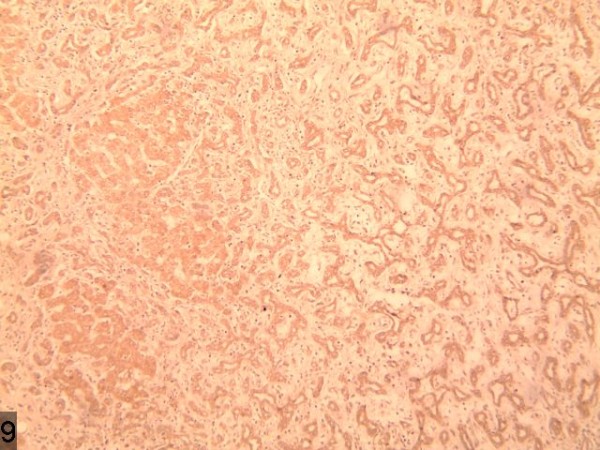
α-fetoprotein stains were weak positive.

**Figure 5:**
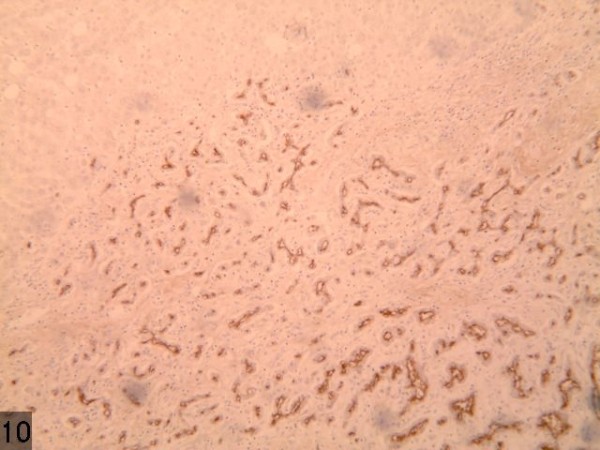
Strong reactivity for epithelial membrane antigen.

The patient, a 51-year-old male, was transferred to receive surgical treatment after he was found to have low density node on the top of his right liver when receiving a chest CT examination during his treatment period on lung infection and cerebral hemorrhage sequelae in the Department of Neurology. The result of his physical examination showed no yellow sclerae or positive sign in his abdomen. The liver function tests were within normal limits. The serum levels of tumor markers, including carcinoembryonic antigen (CEA), carbohydrate antigen (CA) 19-9, and α-fetoprotein (AFP), were not elevated and the hepatitis virology examination was negative. Enhanced CT scanning showed low density nodes in his left hepatic lobe near the top of the liver and an obvious enhancement of the arterial phase, along with which there were also the feeding arteries and perifocal hypertransfusion with an enhanced drop of venous phase and delayed phase. Therefore, the left hepatic nodule was clinically diagnosed with the possibility of liver cancer. When he was receiving an exploratory laparotomy under general anesthesia, the patient was also found to have a medium hard mass measuring about 1.4 cm close to diaphragmatic dome in his left hepatic lobe, but the mass had not infiltrated the liver capsule. The lump was then completely removed together with the margins at a periphery of 2 cm. The pathological report showed bile duct adenoma (BDA) and fatty degeneration of hepatic cells, with the immunohistochemical analysis revealing p53 (−), cytokeratin (CK) 7 (+), CK19 (+), CEA (−), AFP (+), and epithelial membrane antigen (EMA) (+) (Figures 1, 2, 3, 4 and 5).

## Discussion

### Diagnosis

BDA is a rare case of benign intrahepatic tumor which is mostly found in patients between the age of 20 to 70 years old, averaging 55 years old, with no significant gender difference in the incidence of this disease [1]. As the patients often do not present with clinical symptoms, this tumor is usually discovered by accident during a physical examination or an autopsy. It is the same case with the patient of this study: he was found to have liver occupying lesions unexpectedly when undergoing a chest CT examination.

#### Imaging diagnosis

**Figure 6:**
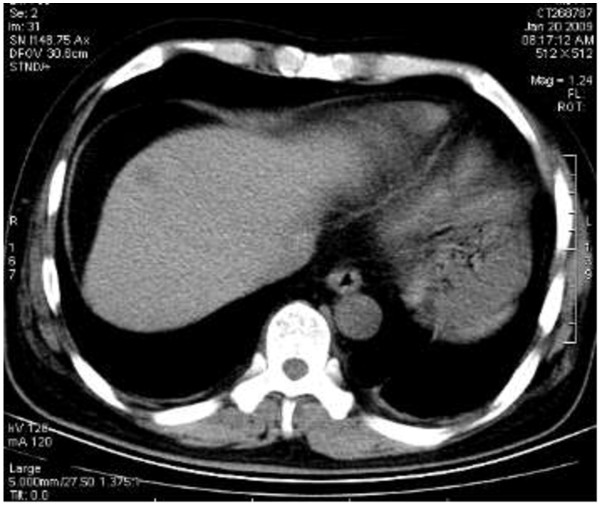
Low density nodule with hazy border close to the liver capsule in the front part of left liver shown by a plain CT scan.

**Figure 7:**
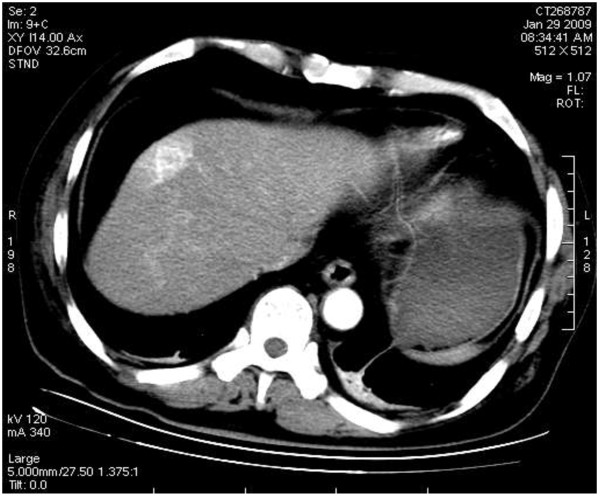
The enhanced uneven nodule of the lesion in arterial phase with an enhanced ring-like peripheral shadow and congestion around the lesion.

**Figure 8:**
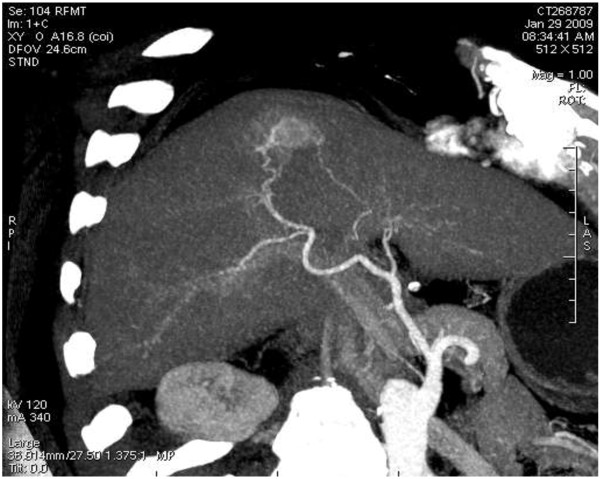
Maximum intensity projection reconstruction showing the lesion blood supply by the branch of right hepatic artery.

**Figure 9:**
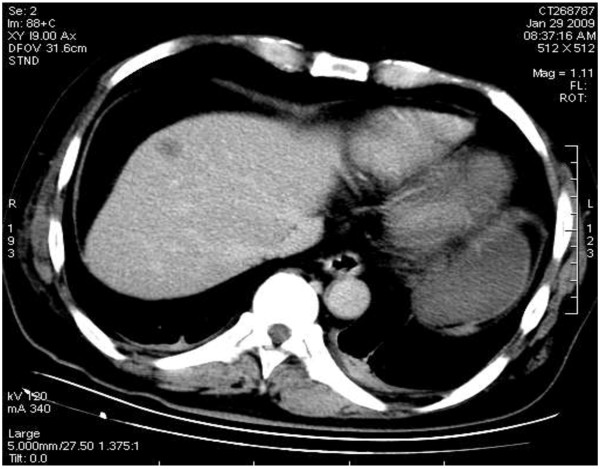
Low density shadow of nodule of the lesion in the portal phase.

As the imaging features of BDA are lacking in specificity, it is very hard to distinguish BDA from primary liver cancer. In this case, a plain CT scan showed that there was a nodular hypodense shadow of 1.5 cm with a hazy border close to the liver capsule in the juncture of segment 4 and 8 (Figure 6). The heterogeneity of the enhanced arterial phase was more intense; an abnormally enhanced ring-like peripheral shadow was also observed. A slightly or moderately enhanced flake-like change was identified in the hepatic tissue around the lesion, which, through maximum intensity projection and volume reconstruction, was shown to get blood supplied by the branch of the right hepatic artery (Figure 7 and Figure 8) and the lesions of both the portal and delayed phases showed a change of low density (Figure 9). The BDA lesions in the 6 cases reported by scholars such as Tajima were detected in the peripheral portion of the liver [2], and 5 of these took the form of hypervascular tumor, which are in accordance with the case we are studying. The CT enhancement method used in our case, however, is different from those in the cases mentioned above. This case being studied was initially misdiagnosed as hepatocellular carcinoma through a preoperative CT scan, the reason for which is that the CT scan demonstrated a typical “quick wash-in and wash-out” sign with an obvious nodular enhancement in the arterial phase of the lesion and low density change in the portal and delayed phases, all of which made it difficult for accurate diagnosis. In a report on BDA by Semelka et al., BDA appeared hypointense on T1-weighted images and hyperintense on T2-weighted images through MRI [3]. However, in the report by Maeda et al., T_2_WI was considered as hypointense; it was proposed that the difference is caused by the number of the fibrous tissues in the tumor and whether there is calcification in the center of the tumor [4].

#### Pathologic diagnosis

**Figure 10:**
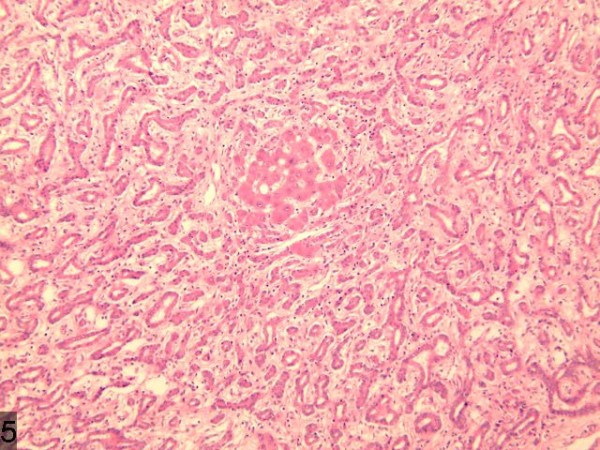
A large quantity of proliferated bile ducts with considerable inflammatory cell infiltration can be observed.

Traditionally speaking, BDA is a type of proliferative lesion originating from the damaged intrahepatic bile duct epithelium [5]. The main features of the pathology of BDA are as follows: i) The lesion is comparatively small, with a diameter between 1 to 20 mm. ii) The lesion is always detected under the hepatic capsule, usually taking the form of solitary nodule, occasionally of multiple nodules, with clear borders but without capsule. iii) The majority of the proliferated bile ducts are observed in the lesion, accompanied by plenty of chronic inflammatory cell infiltration; the epithelial cells of the proliferated bile ducts are well differentiated without obvious atypia. iv) As the disease progresses, the number of proliferated bile ducts and the infiltrated inflammatory cells gradually decreases, while that of fibrous tissues increases. In the late stage, the hyaline-degenerated collagen fibers occupy the region, with only a small amount of bile component and occasionally accompanied with calcification [6]. v) Immunohistochemical stains for CK7, CK19, and CD56 are positive, whereas for Ki67 and p53, stains are negative. In the present case, a large quantity of proliferated bile ducts can be observed, which are lined with single cuboidal epithelial cells with considerable inflammatory cell infiltration and hepatic steatosis (Figure 10), in accordance with the above pathological characteristics. In this case, the weakly positive immunohistochemical AFP and normal serum AFP show no significance in differentiating malignant tumors. Some scholars suggest that the lesion originates from a hamartoma of the glandular components around the bile ducts, and this lacking in atypical and gland mutation both contribute significantly to diagnosis. Further, the variation of the hyaline cell formation is very similar to renal-cell carcinoma. However, scholars, such as Saavedra [7], believe that the positive CK7, EMA, and CEA stains and negative Ki67 and CK20 stains can be helpful for differentiation.

### Treatment

Although BDA is defined as a benign tumor, it still has the possibility of carcinogenesis. Hasebe et al. reported a case in which BDA developed into cholangiocarcinoma [8]. Through imaging examination, it is very hard to distinguish BDA from hepatocellular carcinoma definitely. Even when conducting a frozen section examination, it is still difficult to identify it from cholangiocarcinoma or liver metastasis. Therefore, surgical resection is still needed as a way of treatment and local excision is also feasible. Following surgical resection or local excision, patients have a good prognosis, and so far there is no report about tumor recurrence in the literature.

## Conclusions

BDA is an extremely rare benign tumor, which is difficult to distinguish BDA from hepatocellular carcinoma through imaging examination .Its main character of pathological is a large quantity of proliferated bile ducts, which can be observed in the lesion,They are CK7, CK19, and EMA immunoreactive, and are p53 and Ki67 negative. Surgical resection is needed as a way of treatment.

## Consent

Written informed consent was obtained from the patient for publication of this case and for the accompanying images.

## Competing interests

All authors have made substantive contributions to the study, and are in agreement with the conclusions of the study. Furthermore, there are no financial competing interests.

## Authors’ contributions

LC and FC treated the patient. LC wrote the manuscript. MYX performed the literature search. All authors read and approved the final manuscript.

## Acknowledgement

None.

## References

[B1] Allaire GS Rabin L Ishak KG Sesterhenn IA Bile duct adenoma. A study of 152 cases Am J Surg Pathol 1988 12 708 715 10.1097/00000478-198809000-00007 3046396

[B2] Tajima T Honda H Kuroiwa T Yoshimitsu K Irie H Aibe H Taguchi K Shimada M Masuda K Radiologic features of intrahepatic bile duct adenoma: a look at the surface of the liver J Comput Assist Tomo 1999 23 5 690 695 10.1097/00004728-199909000-00008 10524847

[B3] Semelka RC Hussain SM Marcos HB Woosley JT Biliary hamartomas: solitary and multiple lesions shown on current MR techniques including gadolinium enhancement J Magn Reson Imaging 1999 10 196 201 10.1002/(SICI)1522-2586(199908)10:2<196::AID-JMRI14>3.0.CO;2-R 10441025

[B4] Maeda E Uozumi K Kato N Akahane M Inoh S Inoue Y Beck Y Goto A Makuuchi M Ohtomo K Magnetic resonance findings of bile duct adenoma with calcification Radiat Med 2006 24 459 462 10.1007/s11604-006-0044-z 16958429

[B5] JIang C Che J A case of intrahepatic bile duct adenoma J Diagn Pathol 1998 5 2 117

[B6] Zhang L Xiang L Zhou Q Zhou SQ The clinical and pathological characteristics of intrahepatic bile duct adenoma Chin J Hepatobiliary Surg 2002 8 8 512

[B7] Jorge A-S Hoang MP Murakata LA A typical bile duct adenoma, clear cell type Am J Surg Pathol 2001 25 7 956 960 10.1097/00000478-200107000-00016 11420469

[B8] Hasebe T Sakamoto M Mukai K Kawano N Konishi M Ryu M Fukamachi S Hirohashi S Cholangiocarcinoma arising in bile duct adenoma with focal area of bile duct hamartoma Virchows Archiv 1995 426 209 213 7757293 10.1007/BF00192644

